# Parathyroid Adenoma With Respiratory-Like Epithelium: Case Report of a Potential Mimic With Unknown Etiology

**DOI:** 10.3389/fendo.2021.724766

**Published:** 2021-08-04

**Authors:** C. Christofer Juhlin, Jan Zedenius

**Affiliations:** ^1^Department of Oncology-Pathology, Karolinska Institutet, Stockholm, Sweden; ^2^Department of Pathology and Cytology, Karolinska University Hospital, Stockholm, Sweden; ^3^Department of Molecular Medicine and Surgery, Karolinska Institutet, Stockholm, Sweden; ^4^Department of Breast, Endocrine Tumors and Sarcoma, Karolinska University Hospital, Stockholm, Sweden

**Keywords:** respiratory, epithelium, case report, parathyroid, adenoma

## Abstract

Parathyroid adenoma is a tumor composed of increased parenchymal tissue, often built-up by chief cells, transitional cells or oncocytic cells arranged in acinar or solid formations. Occasionally, rare histological patterns are reported, including cystic or trabecular arrangements. We present a 47 year-old male patient with primary hyperparathyroidism who underwent focused parathyroidectomy for a right inferior adenoma. Surgery was uneventful, but histologically, normal parathyroid tissue adjacent to a tumorous structure displaying a cystic growth pattern was detected. The cells lining the cyst walls appeared cylindrical and pseudo-stratified, vaguely reminiscent of a respiratory type of epithelium usually associated to branchial cleft cysts or thyroglossal cyst remnants, albeit with a tumorous appearance. The respiratory-like epithelium stained positive for parathyroid markers PTH and GATA3, thereby confirming them as parathyroid-derived. The patient was cured from surgery as he displayed normal calcium and PTH levels postoperatively, and is currently alive and well without signs of relapse 4 years after surgery. This is to our knowledge the first report of a parathyroid tumor displaying a respiratory-like epithelium. Experimentally, canine parathyroid glands can develop ciliated respiratory epithelium in response to inhalation of ozone. Our patient is a construction worker with a hypothetically increased risk of continuous ozone exposure. Although this association remains purely speculative, future investigations of this tumor phenotype could perhaps yield novel insights regarding the frequency of this histological variant, potential clinical associations, and clues regarding influencing factors.

## Introduction

Parathyroid adenomas are benign, parenchymal tumors that often secrete parathyroid hormone (PTH), causing hypercalcemia (1). The most common presentation is uniglandular disease in a patient with primary hyperparathyroidism. The acinar component is usually composed of chief cells, transitional cells or oncocytic cells, although rare histological variants such as cystic adenomas, water-clear cell adenomas and lipoadenomas have been described (2–6). In some instances, parathyroid tissue may present in ectopic locations as a consequence of aberrant embryological migration during the fetal stage, and in some instances also present as a component of a branchial cleft anomaly (7).

The parathyroid chief cells, the main cell type of most parathyroid adenomas, are usually polygonal with a neutral to slight eosinophilic cytoplasm, and the nuclei are often round with little atypia (2). In normal parathyroid tissue, the cells are usually arranged in delicate palisading cords, whereas the growth pattern in adenomas usually tend to be acinar or diffuse. Even though the chief cell nuclei often are enlarged in adenomas compared to normal parathyroid tissue, the cells retain their polygonal shape and do not exhibit metaplastic features (2). In this case report, we describe a parathyroid adenoma with a cystic growth, lined by a cell component highly reminiscent of respiratory type epithelium, and describe the histological, expressional and clinical features of this exceedingly unusual manifestation.

## Case Description

**Figure 1:**
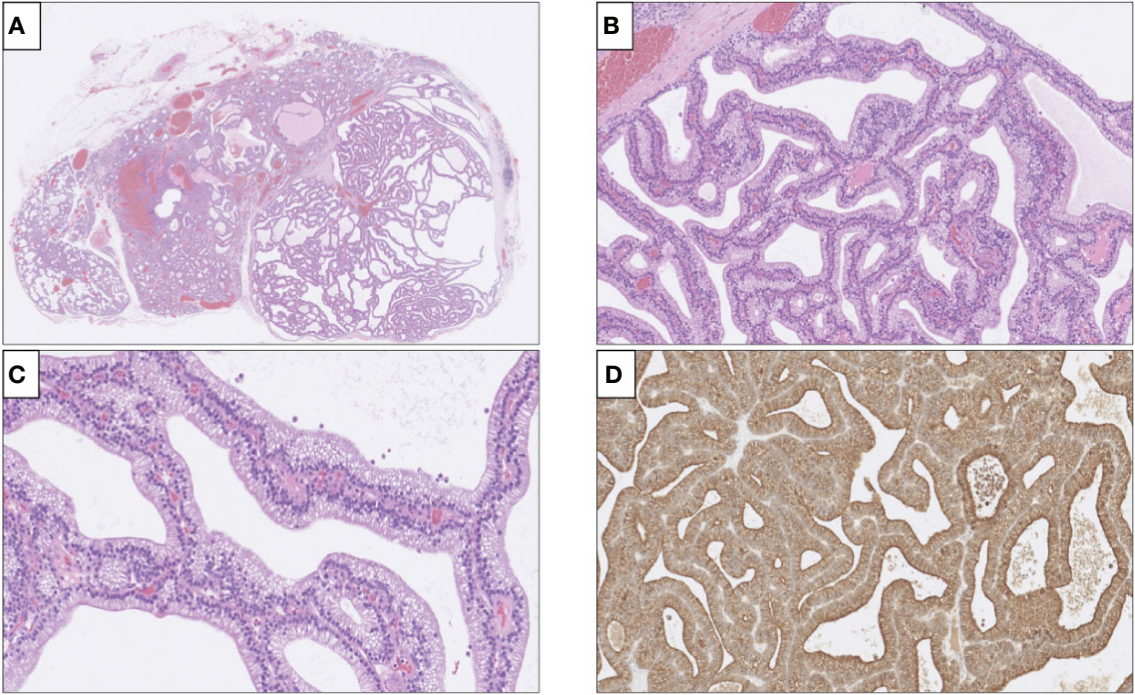
Histological and immunohistochemical attributes of the parathyroid adenoma with respiratory-like epithelium. **(A)** Hematoxylin-eosin (H&E) stain of the excised lesion depicting a cystic appearance (right), with normal rim of parathyroid tissue to the left. Magnification x20. **(B, C)** Cells were pseudo-stratified and cylindrical with palisading nuclei and, appearing focally, processes reminiscent of apical cilia, magnification x200 and x400 respectively. **(D)** Diffuse and strong PTH immunoreactivity was noted.

The patient was a 47 year-old male of Swedish ethnicity without previous medical history. He had no family history suggestive of parathyroid disorders. He worked as a construction worker, did not smoke and only reported moderate use of alcohol. In 2017, he developed fatigue and was subsequently diagnosed with hypercalcemia with an ionized calcium of 1.52 mmol/L (reference 1.15-1.33). His plasma PTH levels were also elevated at 18 pmol/L (reference: 1.6-6.0). The patient was referred to our department for a surgical consult. Clinical examination showed no palpable neck mass. Neck ultrasound identified a 10 mm enlarged right inferior parathyroid gland. No further localization study was performed. The patient was planned for a focused parathyroidectomy, which was performed a month later. The excised gland weighed 700 mg and measured 19 mm. Upon macroscopic examination, the tumor exhibited a cystic appearance. Histologically, the lesion demonstrated a multilobar, cord-like cystic growth pattern, with a single-lined, pseudostratified epithelium built-up by cylindrical cells with basal-positioned nuclei and vacuolated cytoplasm (**Figure 1**). Focally, structures reminiscent of cilia were noted. Tumor nuclei displayed a compact chromatin, and atypia was generally absent. Small foci of infiltrating lymphocytes were also detected, as was an adjacent rim of normal parathyroid tissue. There was no thymic or thyroid tissue present.

Immunohistochemistry was performed, and the cylindrical cells displayed diffuse positivity for the AE1/AE1 pan-keratin cocktail, PTH, GATA binding protein 3 (GATA3) and polyclonal Paired box 8 (PAX8), thereby verifying them as parathyroid-derived (**Figure 1**). Stains for Thyroid transcription factor 1 (TTF1) and Napsin A were negative. To rule out malignant potential, parafibromin and Adenomatous polyposis coli (APC) stains were ordered, and both showed widespread positivity. The Ki-67 proliferation index was determined to 2% by manual counting of 500 cells using an ocular grid. A periodic acid-Shiff (PAS) staining displayed magenta-colored intracytoplasmatic droplets that were absent on a subsequent PAS diastase stain, indicating that these droplets were glycogen-derived. The final diagnosis was a parathyroid adenoma.

Immediately postoperatively, the patient’s PTH levels decreased, indicating cure (1.3 pmol/L), and he was discharged the same day. Subsequent ionized calcium measurements 3 weeks later showed normalized levels at 1.25 mmol/L, and values remains normal at follow-up, although PTH levels one year postoperatively was just above normal range, probably secondary to a synchronous vitamin D deficiency at the time. The patient is well and without any related symptoms as of May 2021.

## Discussion

We report a parathyroid adenoma with a highly unusual growth pattern, characterized by cysts lined by a respiratory-like epithelium. The parathyroid origin was proven by immunohistochemistry, and the functionality of the lesion was also established as surgical removal cured the patient from hypercalcemia. The finding is of potential value for practicing endocrine pathologists as a novel differential diagnosis when investigating cystic lesions lined by a ciliated, cylindric epithelium in the neck.

Usually, the finding of cystic structures lined with cylindrical epithelium in the neck could lead the pathologist to suspect a thyroglossal duct cyst (8). However, the index lesion was tumorous in nature, was not located along the midline, lacked association to the hyoid bone and there was no thyroid tissue within the excised specimen. Moreover, rare cases of third branchial cleft cyst anomalies can present with parathyroid tissue, but the lesion presented herein did not contain thymic tissue, and the respiratory epithelium lining branchial cleft cysts are not immunoreactive to PTH or GATA3 (9). Therefore, the lesion was indeed a parathyroid adenoma displaying a morphology reminiscent of airway epithelium. As no fresh-frozen tissue was kept from the tumor, we could not perform electron microscopy to investigate the suspected cilia component more closely, but previous ultrastructural studies in parathyroid tumors may support the notion that subsets of cases may exhibit ciliated structures (10).

When searching online repositories for similar findings, no credible descriptions of this phenomenon was identified, and the incidence of this type of parathyroid tumor is therefore assumed to be exceedingly low. On an experimental basis, ozone exposure in dogs has been found to prompt the development of ciliated cystic structures within the parathyroid glands (10, 11). Even though the index patient is a construction worker (a profession associated to ozone exposure), we can neither rule out nor confirm that exposure to this substance in any way influenced the development of a parathyroid tumor with respiratory-like epithelium (12). Therefore, it is currently not known if our observations indeed indicate a metaplastic transformation involving parathyroid cells. From a prognostic perspective, the patient did not display persistent or recurrent hypercalcemia, neither were there any signs of atypia in the excised tumor. Retained parafibromin and APC expression in any parathyroid tumor also argues against malignant potential.

To conclude, we describe a parathyroid adenoma with a previously unreported histological growth pattern. Future retrospective analyses of large tumor series can possibly uncover similar cases in which the cellular features were initially overlooked.

## Data Availability Statement

The original contributions presented in the study are included in the article/supplementary material. Further inquiries can be directed to the corresponding author.

## Ethics Statement

Ethics approval no. 01-133 was obtained by KI Forskningsetikkommitté Nord. The patients/participants provided their written informed consent to participate in this study. Written informed consent was obtained from the individual(s) for the publication of any potentially identifiable images or data included in this article.

## Author Contributions

CCJ conceived the idea, reviewed the histology and drafted the manuscript. JZ reviewed the clinical history and edited the manuscript. All authors contributed to the article and approved the submitted version.

## Funding

CCJ is a Junior Clinical Investigator sponsored by the Swedish Cancer Society.

## Conflict of Interest

The authors declare that the research was conducted in the absence of any commercial or financial relationships that could be construed as a potential conflict of interest.

## Publisher’s Note

All claims expressed in this article are solely those of the authors and do not necessarily represent those of their affiliated organizations, or those of the publisher, the editors and the reviewers. Any product that may be evaluated in this article, or claim that may be made by its manufacturer, is not guaranteed or endorsed by the publisher.
